# The Involution of the Uterus and Its Influence on Practical Therapy

**Published:** 1915-01-16

**Authors:** 


### THE INVOLUTION OF THE UTERUS AND ITS INFLUENCE ON PRACTICAL THERAPY.

It has long been known that during the latter period of pregnancy much less total nitrogen is excreted in the urine than in other women taking similar diet and similarly environed. At the time of labour the nitrogen excretion is even lower still; while during the puerperium it rises to considerably above the normal level, and this high excretion persists for about two weeks. This latter phenomenon has evoked several more or less ingenious hypothetical explanations. One author attributed it to lactation, basing his belief on the hypothesis that the fat of milk is derived from protein. Unfortunately for this theory, the nitrogen excretion is just as high in those who do not suckle as in those who do; and in any case it fails to explain the return to normal excretion at the end of a fortnight. Another idea was that starvation had something to do with the cause of the facts; but it was shown that women who were liberally fed from the time of delivery excreted just as much nitrogen as those who were kept on low diet for a few days.

A more credible theory than either of these attributes the nitrogen excretion to an autolysis of the muscles tissue of the enlarged uterus, which within a few weeks of delivery is reduced in weight from about 1,000 down to 50 or 60 grammes. Professor J. M. Slemons,* of the University of California, has taken the opportunity of testing this hypothesis by observations on cases of Cæsarean section. By comparing the nitrogen excretion following conservative section with that following section with supravaginal hysterectomy, he is able to arrive at certain definite conclusions in favour of uterine involution as the cause of the increased nitrogen excretion in the early weeks of the puerperium. He finds that the amount of excess elimination of nitrogen corresponds to the nitrogenous content of the non-involuted uterus. Evidence of this is derived from comparison of the puerperal and post-puerperal periods when the uterus has not been removed; from comparison of the aggregate excretion in cases in which the uterus was removed and in which it was not; and from comparison of the actual daily difference in such cases with the theoretical amount of nitrogen which would be expected as a result of the involutionary process. It is concluded that waste products from the involuting uterus pass into the circulation, are excreted by the kidneys, and in some measure throw additional work upon these organs during the early part of the puerperium.

It does not follow, says Professor Slemons, that the diet should be restricted in the puerperium. On the contrary, provided the patient is normal, the increase in the excretion of nitrogen is not large enough to indicate any need for restriction of protein intake. If any therapeutic indication can be inferred, it is in the nature of a justification of the present-day tendency to allow a generous diet to recently delivered women.

On the other hand, in patients suffering from eclampsia or from any lesser manifestations of the toxæmia of pregnancy, any additional work thrown upon the kidneys during the puerperium does become a matter of practical importance. In such circumstances these organs should be given the fullest opportunity to recover from the strain to which they have been subjected, and with this end in view the diet should be limited. Such precaution is necessary, not only because the renal cells have been damaged, but also because an excretory capacity somewhat greater than normal is desirable.

* *Johns Hopkins Hospital Bulletin*, July 1914, p. 195.

